# Tau Positron Emission Tomography and Neurocognitive Function Among Former Professional American-Style Football Players

**DOI:** 10.1089/neu.2022.0454

**Published:** 2023-08-16

**Authors:** Maeva Dhaynaut, Rachel Grashow, Marc D. Normandin, Ona Wu, Dean Marengi, Douglas P. Terry, Justin S. Sanchez, Marc G. Weisskopf, Frank E. Speizer, Herman A. Taylor, Nicolas J. Guehl, Sudha Seshadri, Alexa Beiser, Daniel H. Daneshvar, Keith Johnson, Grant L. Iverson, Ross Zafonte, Georges El Fakhri, Aaron L. Baggish

**Affiliations:** ^1^ Gordon Center for Medical Imaging, Department of Radiology, Massachusetts General Hospital, Harvard Medical School, Boston, Massachusetts, USA.; ^2^ Department of Environmental Health, Harvard T. H. Chan School of Public Health, Boston, Massachusetts, USA.; ^3^ Football Players Health Study at Harvard University, Harvard Medical School, Boston, Massachusetts, USA.; ^4^ Athinoula A. Martinos Centre for Biomedical Imaging, Department of Radiology, Massachusetts General Hospital, Charlestown, Massachusetts, USA.; ^5^ Vanderbilt Sports Concussion Center, Department of Neurological Surgery, Vanderbilt University Medical Center, Nashville, Tennessee, USA.; ^6^ Division of Nuclear Medicine and Molecular Imaging, Department of Radiology, Massachusetts General Hospital, Harvard Medical School, Boston, Massachusetts, USA.; ^7^ Channing Division of Network Medicine, Brigham and Women's Hospital, Harvard Medical School, Boston, Massachsetts, USA.; ^8^ Morehouse School of Medicine, Atlanta, Georgia, USA.; ^9^ The Glenn Biggs Institute for Alzheimer's and Neurodegenerative Diseases, UTHSA, San Antonio, Texas, USA.; ^10^ NHLBI's Framingham Heart Study, Framingham, Massachusetts, USA.; ^11^ Department of Biostatistics and Neurology, Boston University School of Public Health, Boston, Massachusetts, USA.; ^12^ Department of Physical Medicine and Rehabilitation, Spaulding Rehabilitation Hospital, Charlestown, Massachusetts 02129, USA.; ^13^ Department of Physical Medicine and Rehabilitation, Harvard Medical School, Charlestown, Massachusetts, USA.; ^14^ Department of Physical Medicine and Rehabilitation, Schoen Adams Research Institute at Spaulding Rehabilitation, Charlestown, Massachusetts, USA.; ^15^ Sports Concussion Program, MassGeneral Hospital for Children, Boston, Massachusetts, USA.; ^16^ Cardiovascular Performance Program, Massachusetts General Hospital, Boston, Massachusetts, USA.; ^17^ Department of Cardiology, Lausanne University Hospital (CHUV) and Institute for Sport Science, University of Lausanne (ISSUL), Lausanne, Switzerland.

**Keywords:** concussion, football, FTP, PET, TBI

## Abstract

American-style football (ASF) players experience repetitive head impacts that may result in chronic traumatic encephalopathy neuropathological change (CTE-NC). At present, a definitive diagnosis of CTE-NC requires the identification of localized hyperphosphorylated Tau (p-Tau) after death via immunohistochemistry. Some studies suggest that positron emission tomography (PET) with the radiotracer [^18^F]-Flortaucipir (FTP) may be capable of detecting p-Tau and thus establishing a diagnosis of CTE-NC among living former ASF players. To assess associations between FTP, football exposure, and objective neuropsychological measures among former professional ASF players, we conducted a study that compared former professional ASF players with age-matched male control participants without repetitive head impact exposure. Former ASF players and male controls underwent structural magnetic resonance imaging and PET using FTP for p-Tau and [^11^C]-PiB for amyloid-β. Former players underwent neuropsychological testing. The ASF exposure was quantified as age at first exposure, professional career duration, concussion signs and symptoms burden, and total years of any football play. Neuropsychological testing included measures of memory, executive functioning, and depression symptom severity. P-Tau was quantified as FTP standardized uptake value ratios (SUVR) and [^11^C]-PiB by distribution volume ratios (DVR) using cerebellar grey matter as the reference region. There were no significant differences in [^18^F]-FTP uptake among former ASF players (*n* = 27, age = 50 ± 7 years) compared with control participants (*n* = 11, age = 55 ± 4 years), nor did any participant have significant amyloid-β burden. Among ASF participants, there were no associations between objective measures of neurocognitive functioning and [^18^F]-FTP uptake. There was a marginally significant difference, however, between [^18^F]-FTP uptake isolated to the entorhinal cortex among players in age-, position-, and race-adjusted models (*p* = 0.05) that may represent an area of future investigation. The absence of increased [^18^F]-FTP uptake in brain regions previously implicated in CTE among former professional ASF players compared with controls questions the utility of [^18^F]-FTP PET for clinical evaluation in this population.

## Introduction

Repetitive head impacts (RHI) may increase the risk of adverse long-term cognitive outcomes.^1^ The proposed primary pathology underlying this association is chronic traumatic encephalopathy neuropathological change (CTE-NC), which is characterized by the presence and localization of cortical perivascular hyperphosphorylated Tau (p-Tau).^2^

Recently, clinical criteria have been proposed for the antemortem diagnosis of CTE-NC, but at present, a definitive diagnosis of CTE-NC can only be established post-mortem using immunohistochemical brain examination.^3^ Development of therapeutic strategies to treat people with CTE necessitates accurate tools for antemortem diagnosis. Accordingly, there has been considerable interest in developing tools to diagnose CTE-NC among living persons.^4^ Positron emission tomography (PET) utilizing radiotracers designed to have selective high binding affinity for p-Tau in Alzheimer disease^5^ has been employed recently for this purpose.^6^

The proposed association between RHI and CTE-NC has garnered attention among American-style football (ASF) players and their clinicians.^3^ PET imaging with the radiotracer [^18^F]-Flortaucipir (FTP) has been proposed as a way to detect p-Tau and potentially diagnose CTE-NC among living former ASF players.^7,8^

While pre- and post-mortem imaging with FTP has not universally been shown to confirm conventional postmortem CTE-NC diagnosis,^9,10^ a recent study reported increased cortical FTP uptake among 26 former professional ASF players with subjective neurocognitive complaints compared with age-matched men.^6^ This important finding suggests a clinical rationale for the use of FTP PET among former ASF players and other populations exposed to RHI.^6^

A subsequent *in vivo* study on elite contact sport athletes, cognitively normal controls, mild cognitive impairment patients, and Alzheimer disease patients revealed mildly elevated FTP binding for amyloid-negative patients at risk for CTE-NC, suggesting that FTP may not be appropriate for studies on early-stage CTE-NC.^11^ To date, these findings have yet to be reconciled.

We therefore conducted the present study with two distinct objectives. First, we sought to validate previous findings^6^ in a similar sample of former ASF players using comparable methodology. Second, we hypothesized that an analytical approach to FTP PET data with more precise regional specificity and using an additional concussion signs and symptoms-based football exposure would provide novel insights into the relationship between ASF exposure and p-Tau deposition.

## Methods

### Participants

The Football Players Health Study (FPHS) at Harvard University^12^ recruited 31 former ASF players between the ages of 33 and 60 who: (1) contracted with professional ASF leagues after 1960, the year hard plastic helmets were adopted^13^; (2) completed the FPHS Health and Wellness survey between the ages of 29 and 55 years; and (3) had previously self-reported a diagnosis of cardiometabolic, neurocognitive, chronic pain, and/or sleep disorder or as unafflicted by any of those conditions.^14^

These participants underwent PET scanning on the Discovery MI-5 (GE Healthcare) PET/CT scanner at the Gordon Center for Medical Imaging at Massachusetts General Hospital (Boston, MA) between 2019 and 2020. We examined p-Tau tracer uptake among 27 former professional ASF players after exclusions based on tracer failure, compromised cerebellum data, or lack of corollary MRI data.

A convenience sample of 11 white male controls (age range: 47–60) was recruited from the Framingham Heart Study^15^ cohort to characterize molecular signatures of pre-clinical and clinical Alzheimer disease using PET. These control participants were unexposed to RHI and scanned on the same Discovery MI-5 (GE Healthcare) PET/CT scanner at the Gordon Center for Medical Imaging at Massachusetts General Hospital (Boston, MA) between 2018 and 2020. Informed consent was obtained from all participants and approval was granted by the Human Research Protection Program at Mass General Brigham.

### Covariates

Age and race were determined as described previously.^1^ Only players who endorsed either “Black” or “white” were included, because of the limited ASF participation of other race groups. The FPHS Health and Wellness survey queried age of first football exposure and years of youth, high school, collegiate, and professional play.

These were used to categorize four football exposure measures: (1) age of first football exposure, (2) total years of football, (3) total National Football League (NFL) seasons, and (4) self-reported total number of concussion signs and symptoms (concussion signs and symptoms score) accumulated during football play or practice.

As described previously,^1,16,17^ the concussion signs and symptoms score was quantified by summing the frequency of each of 10 concussion signs and symptoms (included headaches, nausea, dizziness, loss of consciousness, memory problems, disorientation, confusion, seizure, visual problems, and feeling unsteady on one's feet) reported to have occurred after a blow to the head or neck. Total NFL seasons was used as a continuous variable in models adjusting for age, race, and position. Position was divided into defensive back, defensive line, kicker/punter, linebacker, offensive line, quarterback, running back, tight end, wide receiver, and special teams only.

For comparison with the football player sample in Stern and associates,^6^ we conducted sensitivity analyses only in players with subjective cognitive complaints. Consistent with previous work,^6^ perceived neurocognitive function was measured (Quality of Life in Neurological Disorders [Neuro-QOL^18^]-Applied Cognition-General Concerns [short form]). Specifically, to create a subsample of football players selected for reporting subjective neurocognitive dysfunction, participants scoring more than one standard deviation below the mean (i.e., T-score <40) were considered to have subjective cognitive dysfunction, and they were included in sensitivity analyses. An additional sensitivity analysis was conducted in ASF players over the age of 43 to match ASF participants in the Stern and associates^6^ study.

### Neuroimaging data acquisition

#### Magnetic resonance imaging (MRI)

All subjects underwent structural MRI procedures for anatomical reference. A three-dimensional (3D) structural T1-weighted multi-echo magnetization-prepared rapid gradient-echo (MEMPRAGE) image was acquired using a 3 T Skyra (Siemens Medical Systems) scanner and root-mean squared image calculated for the population of former ASF players. A 3D structural T1-weighted turbo field echo (TFE) was acquired using a 3 T Achieva (Philips) scanner for the control population. All MRI scans had a matrix size of 256 × 256 and a slice thickness of 1 mm and 1 mm isotropic voxel dimensions.

#### Positron mission tomography (PET)

All subjects underwent PET procedures on the same scanning equipment in the same location. Each participant received Aβ and p-Tau PET imaging measured by C-Pittsburgh Compound B([^11^C]-PiB) and [^18^F]-FTP (Avid Radiopharmaceuticals), synthesized and administered onsite. [^11^C]-PiB imaging was acquired in dynamic mode with an injection of 15mCi (555 MBq) intravenous bolus, and [^18^F]-FTP was measured 75 min after a 10mCi (370 MBq) bolus injection for a duration of 30 min.

Prior to each PET scan, a low-dose X-ray computed tomography (CT) scan was performed for attenuation correction. Each PET scan used the full width at half maximum (FWHM) spatial resolution, measured at the center of the axial field of view (radial position = 1 cm), was 4.3 mm and 5.1 mm in transverse and axial directions, respectively.

PET data were reconstructed using a validated 3D time-of-flight iterative reconstruction algorithm with five iterations and 16 subsets while applying corrections for scatter, attenuation, deadtime, random coincident events, and scanner normalization. Final reconstructed images contained voxel dimensions of 256 × 256 × 89 and voxel sizes of 1.173 × 1.17 × 2.8mm.

#### Image processing and analysis

PET data processing was performed using Matlab software,^19^ based on code and function from SPM12^20^ and FSL^21^ software packages. The MRI images were processed and manually edited on FreeSurfer (FSv6.0).^22^ All MRI and PET images were registered into the standard Montreal Neurological Institute (MNI) space as described previously.^23^

The PET images were motion-corrected and rigidly co-registered to the structural MRI image, transformed into the MNI space using a 12-parameter affine transformation followed by nonlinear warping. The transformation matrices were combined and applied inversely on MNI, Harvard–Oxford atlases (available in FSL), and FreeSurfer regions to warp the regions of interest (ROI; Supplementary Table S1) into the native PET images for extraction of radioactivity time–activity curves.

The PiB and FTP retentions were expressed by distribution volume ratios (DVR) and 80–100 min standardized uptake value ratios (SUVR), respectively, with the cerebellar grey matter as reference.^23,24^ Subjects were defined as Aβ positive if the PiB DVR global Aβ burden in a large neocortical aggregate (FreeSurfer-derived “FLR” regions composed of frontal, lateral parietal and temporal, and retrospenial regions) was ≥1.2.^25^

The ROIs surveyed were bilateral superior frontal, bilateral medial temporal, and left parietal from the MNI and Harvard–Oxford atlases to compare with published findings.^6^ We additionally utilized FreeSurfer parcellation results to examine more precisely brain regions from the medial temporal cortex known to be involved in AD and CTE-NC,^26^ such as the entorhinal cortex,^27,28^ hippocampus,^29^ parahippocampus, and amygdala.^30^ We calculated correlations between choroid plexus and hippocampal FTP uptake separately in Black and white players to explore evidence of potential off-target neuromelanin FTP binding.

### Neuropsychological Testing

In-person neuropsychological tests were selected to approximate testing conducted by Stern and associates,^6^ including measures of depression (PHQ-9),^31^ memory (Neuropsychological Assessment Battery [NAB] List Learning Test Delayed Recall),^32^ and executive function (Delis-Kaplan Executive Function System Category Fluency Test [D-KEFS]^33^).

The PHQ-9 is a 9-item self-report questionnaire designed to assess symptoms of depression experienced during the previous fortnight.^31^ Participants rate the frequency of depression-related symptoms and/or behaviors on a 0–3 scale, with higher scores representing greater symptoms, and the cutoff score for screening positively for depression is 10.^34,35^ The D-KEFS Category Fluency Test^33^ involves naming as many words that belong to a specific category as possible within one minute (i.e., first names for a specified gender). The number of correct words produced are age-corrected (mean = 10, standard deviation [SD] = 3).

The NAB List Learning Test^32^ involves three learning trials of a 12-word list, followed by an interference list, and then free recall testing immediately after the interference list and after a 10–15 min delay. Participants who obtained a low score on a performance validity test (Test of Memory Malingering or TOMM,^36^ Trial 1 score ≤35) were excluded from analyses using the cognitive test scores, but imaging data and mood scales for these participants were included.

### Statistical analysis

Between group differences were calculated with voxelwise linear regression models of FTP SUVR maps using SPM12 (*p* < 0.005; uncorrected for multiple comparisons) and restricted to ≥100 voxel clusters to increase comparability with similar studies. Between group comparisons for region-based analyses was performed with Mann–Whitney tests. Spearman age-adjusted partial correlations were used to illustrate relationships between regional FTP SUVRs and total years of football exposure.

We utilized a two-step residual method adjusting for age, race, and position^37^ to assess associations between SUVR values and football exposures. First, we used demographic and football-relevant variables from the questionnaire administered to 2265 FPHS participants within the age range of the PET subjects (29 years to 60 years). We extracted the residuals from separate linear regression models of each football exposure predicted by age, race, and football position.

We then tested linear models to predict PET SUVR values using the residuals from the first stage model for each exposure. By design, the residuals reflect the variation in each football exposure variable independent of age, race, and position, thus adjusting for age, race, and position. Age- and race-adjusted multi-variable linear regression was used to measure associations between SUVR and neurocognitive and mood outcomes (the dependent variables). Exposure analyses were conducted using R Statistical Software.^38^

All sensitivity analyses on subsets of the former player sample were conducted and analyzed as described previously. Distributions were reported using means and SD measures, and statistical significance was set at *p* < 0.05.

## Results

### Demographic, amyloid-β, and football-related participant characteristics

Former ASF players (*n* = 27) and control participants (*n* = 11) were 50 ± 7 and 55 ± 4 years of age, respectively (Table 1). All control participants self-identified as white and 48% of former ASF players self-identified as Black. No individual in either group met criteria for amyloid-β positivity. Former ASF players started organized football at age 11 ± 4 years and spent 7 ± 4 years playing at the professional level resulting in an average of 23 ± 3 total football years per ASF participant. The ASF players reported 34.4 ± 37.2 concussion signs and symptoms accumulated during either play or practice.

**Table 1.** Sample Characteristics for Former Football Players and Controls

Characteristic	Former football players (*n* = 27)	Controls (*n* = 11)
Years of age, mean (SD)	50.1 ± 7.1	54.6 ± 3.9
Black race, no. (%)	13 (48.0)	0 (0)
Male, no. (%)	27 (100)	11 (100)
Amyloid-β score (PiB DVR), mean (SD)	1.02 ± 0.04	1.06 ± 0.05
Years played in NFL, mean (SD)	6.6 ± 3.5	-
Total years of football, mean (SD)	23.0 ± 3.2	-
Concussion signs and symptoms summary score, mean (SD)	34.4 ± 37.2	
Age of first exposure in years, mean (SD)	10.7 ± 2.6	-
Neuro-QOL Applied Cognition T-score, mean (SD)	40.3 ± 10.5	-
PHQ-9, mean (SD)	3.9 ± 5.4	-
D-KEFS Category Fluency, mean (SD) (scaled score)	11.3 ± 3.5	-
NAB List Learning Delayed Recall T-score, mean (SD)	45.7 ± 13.4	-

D-KEFS, Delis-Kaplan Executive Function System; NFL, National Football League; PHQ-9, Patient Health Questionnaire-9, a measure of depression; SD, standard deviation.

### Brain atlas-derived FTP uptake

**FIG. 1.**
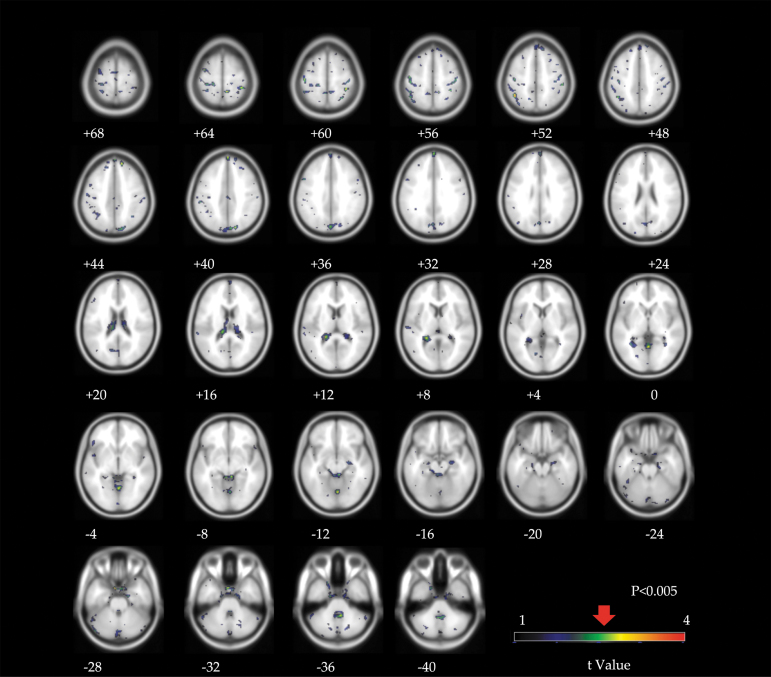
Voxelwise linear regression analysis of Flortaucipir standard uptake value ratio (SUVR) statistic parametrical maps comparing controls and former American-style football (ASF) players. Highlighted voxels indicate higher regional uptake among former players compared to controls. The significance threshold (*p* < 0.005 uncorrected for multiple comparisons) is indicated at the t-value shown in the color bar. Numbers listed below each brain map correspond to the distance (in mm) above (positive) or below (negative) the anterior and posterior commissures. The left hemisphere is shown on the right.

**FIG. 2.**
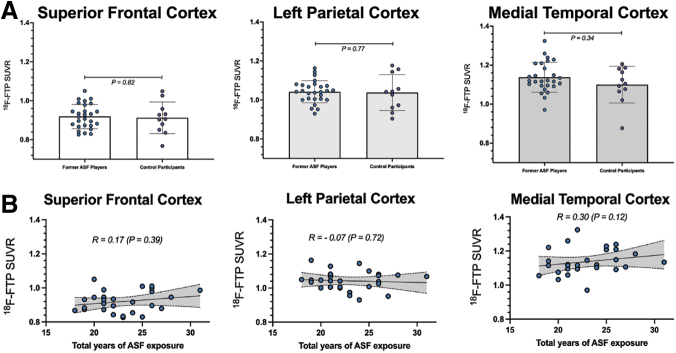
Comparison of regional [^18^F]-Flortaucipir (FTP) standard uptake value ratio (SUVRs) (**A**) Comparison of regional [^18^F]-FTP uptake derived from brain atlas-based analysis between former ASF players (*n* = 27) and controls (*n* = 11) in the superior frontal, left parietal, and medial temporal cortices. The *p* values are derived from Mann–Whitney tests. (**B**) Age-adjusted partial correlations between total years of football play and [^18^F]-FTP uptake in these three brain regions. The *p* values are derived from age-adjusted partial Spearman correlations.

Voxelwise linear regression analyses demonstrated no differences in FTP uptake between former ASF players and controls (Fig. 1; Supplementary Fig. S1). Clusters limited to ≥100 contiguous voxels produced null results. There were effectively no differences in regional tracer uptake between former ASF players and controls in the superior frontal (ASF player SUVR = 0.92 ± 0.06 vs. control SUVR = 0.90 ± 0.09, *p* = 0.55), left parietal (1.04 ± 0.06 vs. 1.04 ± 0.09, *p* = 0.78), and medial temporal (1.14 ± 0.08 vs. 1.09 ± 0.10, *p* = 0.32) brain regions (Fig. 2A). In addition, there were no notable associations between total years of football and SUVR values in these brain regions (Fig. 2B).

Among ASF players, adjusting for age, race, and field position yielded virtually no difference between FTP uptake in each brain region and the age of first football exposure, total years of football exposure, total NFL years, and concussion signs and symptoms score (Table 2A).

**Table 2.** Associations Between [^18^F]-FTP SUVR PET Tracer Uptake and Football Exposures Adjusted for Age, Race, and Position

A. Brain atlas region SUVR	β	95% CI	*p*
Total years of football			
Superior frontal	0.016	-0.016, 0.048	0.31
Left parietal	-0.006	-0.035, 0.024	0.69
Medial temporal	0.016	-0.024, 0.055	0.43
Number of NFL seasons			
Superior frontal	0.011	-0.015, 0.036	0.41
Left parietal	0.003	-0.021, 0.027	0.81
Medial temporal	0.012	-0.02, 0.044	0.43
Age of first exposure			
Superior frontal	0.019	-0.007, 0.044	0.14
Left parietal	0.011	-0.012, 0.035	0.33
Medial temporal	0.003	-0.03, 0.036	0.85
Concussion symptom score			
Superior frontal	-0.02	(-0.049, 0.005)	0.11
Left parietal	-0.01	(-0.031, 0.021)	0.72
Medial temporal	0.01	(-0.026, 0.045)	0.59

B. FreeSurfer region SUVR	β	95% CI	*p*
Total years of football			
Hippocampus	-0.025	-0.088, 0.038	0.43
Amygdala	0.017	-0.022, 0.056	0.38
Entorhinal	0.035	-0.003, 0.074	0.07
Parahippocampus	0.013	-0.016, 0.042	0.36
Number of NFL seasons			
Hippocampus	-0.006	-0.057, 0.045	0.81
Amygdala	0.017	-0.014, 0.047	0.28
Entorhinal	0.031	0.001, 0.061	0.05
Parahippocampus	0.019	-0.003, 0.042	0.09
Age of first exposure			
Hippocampus	-0.009	-0.062, 0.043	0.72
Amygdala	0.016	-0.015, 0.047	0.29
Entorhinal	0.029	-0.002, 0.059	0.07
Parahippocampus	0.019	-0.003, 0.042	0.09
Concussion signs and symptom score			
Hippocampus	0.004	(-0.053, 0.061)	0.893
Amygdala	0.015	(-0.019, 0.049)	0.371
Entorhinal	0.008	(-0.028, 0.044)	0.667
Parahippocampus	-0.011	(-0.037, 0.015)	0.378

Parameter estimates and confidence intervals are in units of standard deviation for both brain atlas-derived (**A**) and FreeSurfer derived (**B**) estimates (*n* = 27). CI, confidence interval; NFL, National Football League.

### FreeSurfer-derived FTP uptake

**FIG. 3.**
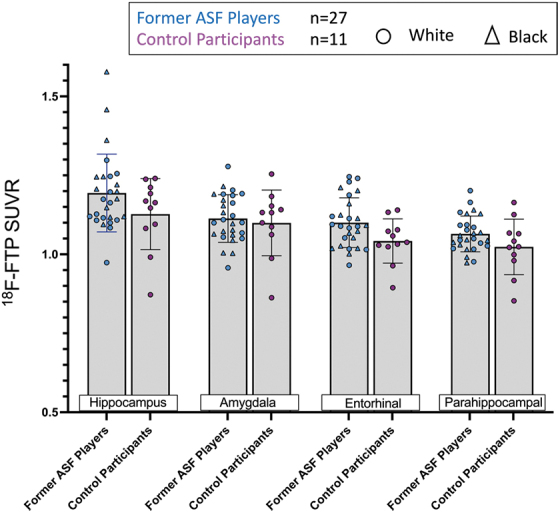
Comparison of American-style football (ASF) exposure and [^18^F]-Flortaucipir (FTP) standard uptake value ratio (SUVRs) in medial temporal subregions. Comparison of regional [^18^F]-FTP uptake derived from FreeSurfer-based analysis between controls and ASF players in the hippocampus, amygdala, entorhinal cortex, and parahippocampus. Triangles indicate Black former ASF players and circles indicate white former ASF players.

To further examine regional FTP uptake specificity, we utilized FreeSurfer to measure between-group differences in discrete sections of the medial temporal cortex because this region contained the highest overall uptake in regional atlas-based analyses. The FTP uptake among former ASF players was comparable to controls in the entorhinal cortex (former ASF player SUVR = 1.10 ± 0.08 vs. control SUVR = 1.04 ± 0.07, *p* = 0.10), parahippocampus (1.06 ± 0.06 vs. 1.02 ± 0.09, *p* = 0.16), hippocampus (1.19 ± 0.12 vs. 1.13 ± 0.11, *p* = 0.26), and amygdala (1.11 ± 0.08 vs. 1.10 ± 0.10, *p* = 0.92; Fig. 3).

The relationships between brain region-specific FTP uptake and professional ASF exposure are shown in Figure 3. Among former ASF players after adjustment for age, race, and position, there was a marginally significant association between NFL career duration and FTP uptake in the entorhinal cortex (β = 0.031; 95% confidence interval [CI] = 0.001-0.061, *p* = 0.05; Table 2B).

### Neuropsychological testing results

Four participants were excluded from analyses of the cognitive test scores because of obtaining low scores on performance validity testing. There were no notable associations between neuropsychological testing and brain atlas-derived FTP uptake in the three interrogated brain regions (Table 3A). The FTP uptake was not associated with objective neurocognitive testing (semantic verbal fluency and list learning memory) or depression symptoms in the three atlas-derived interrogated brain regions or the four FreeSurfer-derived brain regions (Table 3).

**Table 3.** Associations Between [^18^F]-FTP Uptake and Neuropsychological and Neurocognitive Assessments

	Depression (PHQ-9)* n* = 27			Category fluency (D-KEFS)* n* = 23			Delayed recall (NAB List Learning)* n* = 23		
A. Brain atlas SUVR	β	95% CI	*p*	β	95% CI	*p*	β	95% CI	*p*
Superior frontal	-0.017	-2.219, 2.186	0.99	-0.806	-2.45, 0.838	0.32	-3.333	-8.942, 2.277	0.23
Left parietal	-1.03	-3.31, 1.251	0.36	0.231	-1.423, 1.885	0.77	1.558	-4.116, 7.232	0.57
Medial temporal	0.948	-1.238, 3.134	0.38	-0.742	-2.524, 1.039	0.40	0.266	-6.007, 6.539	0.93

B. FreeSurfer SUVR	β	95% CI	*p*	β	95% CI	*p*	Β	95% CI	*p*
Hippocampus	-1.306	-3.702, 1.09	0.27	-0.153	-1.87, 1.564	0.85	2.69	-3.102, 8.483	0.34
Amygdala	-0.091	-2.329, 2.146	0.93	-0.091	-1.765, 1.583	0.91	1.989	-3.714, 7.691	0.47
Entorhinal	-0.024	-2.213, 2.166	0.98	-0.147	-1.747, 1.453	0.85	1.817	-3.643, 7.278	0.50
Parahippocampus	0.013	-2.179, 2.205	0.99	-0.143	-1.747, 1.461	0.85	3.991	-1.213, 9.195	0.13

Age- and race-adjusted associations between brain atlas (**A**) and FreeSurfer-based (**B**) SUVR PET tracer uptake and neuropsychological test performance. SUVR parameter estimates are shown as scaled per standard deviation of the SUVR. CI, confidence interval; SUVR, standardized uptake value ratios.

### Sensitivity analyses

**FIG. 4.**
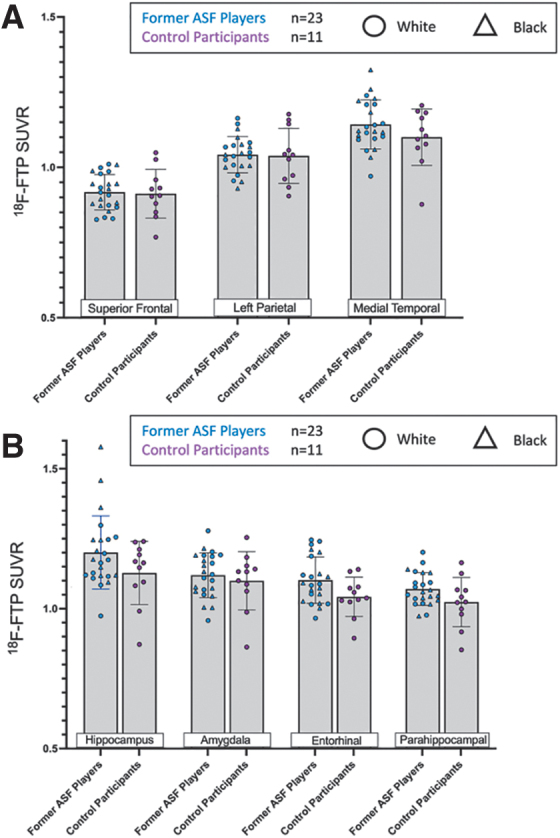
Age-restricted [^18^F]-Flortaucipir (FTP) uptake analysis. (**A**) comparison of regional [^18^F]-FTP uptake derived from brain atlas-based analyses in the superior frontal, left parietal and medial temporal cortex. (**B**) FreeSurfer-based analysis comparing controls and American-style football (ASF) players in the hippocampus, amygdala, entorhinal cortex, and parahippocampus. Triangles indicate Black former ASF players and circles indicate white former ASF players.

**FIG. 5.**
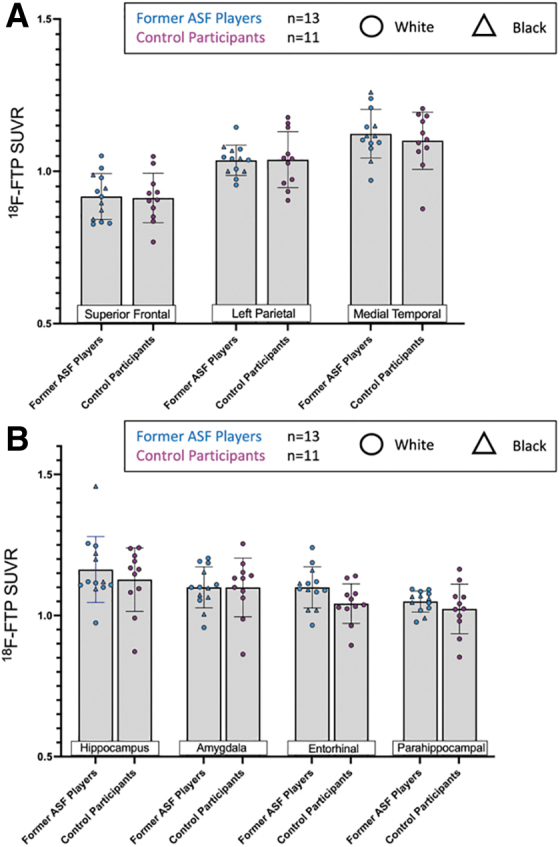
[^18^F]-Flortaucipir (FTP) uptake analysis restricted to American-style football (ASF) players reporting subjective neurocognitive complaints. (**A**) comparison of regional [^18^F]-FTP uptake derived from brain atlas-based analyses in the superior frontal, left parietal, and medial temporal cortex. (**B**) FreeSurfer-based analysis comparing controls and ASF players in the hippocampus, amygdala, entorhinal cortex, and parahippocampus. Triangles indicate Black former ASF players and circles indicate white former ASF players.

We conducted two sensitivity analyses that selected former ASF player participants to align with a previous study.^6^ We examined brain atlas and FreeSurfer-derived FTP uptake among the subset of relatively older former players (≥ 44 years). Separately, we selected players with significant self-reported cognitive problems (*n* = 13; Neuro-QOL <40). We found similar null results with these subsamples as we did in the full analysis (Fig. 4, 5**)** in that the analyses of the cognitive test scores and the depression measure showed no significant associations with SUVR values in the regions evaluated with brain atlas or FreeSurfer as in the full group (data not shown).

### Additional analyses

Correlations between choroid plexus and hippocampal FTP uptake were statistically significant and stronger in Black former ASF players (R^2^ = 0.42, *p* = 0.016) compared with white former ASF players (R^2^ = 0.26, *p* = 0.064), suggesting the possible contribution of race-associated off-target binding of FTP to neuromelanin.

## Discussion

This study was designed to examine p-Tau deposition among former professional ASF players using FTP PET. We detected essentially no differences between FTP uptake among former ASF players and age-matched controls using both brain atlas-derived analyses and a more precise FreeSurfer ROI evaluation. We saw no relationship between FTP uptake and performance on objective neuropsychological testing in primary or sensitivity analyses.

FreeSurfer-derived ROI analyses revealed a marginally significant relationship between duration of NFL career and FTP uptake within the entorhinal cortex after adjustment for age, race, and position, an area in which p-Tau deposition has been associated with cognitive aging and the emergence of neurodegenerative disease.^39,40^ Results across all analyses were similar when we limited the player cohort to older players and separately to players with subjective cognitive complaints.

The CTE-NC is a neuropathological entity that can only be identified after death using immunohistochemistry and microscopic examination of brain tissue. New consensus criteria for traumatic encephalopathy syndrome were published in 2021, and researchers can now begin to determine whether post-mortem CTE-NC is associated with mild cognitive impairment, neurobehavioral dysregulation, or dementia during life.^41^

A recent study found increased FTP uptake in several brain regions among former ASF players with subjective neurocognitive complaints compared with a small sample of non-football playing controls,^6^ while another showed higher frontotemporal FTP binding in amyloid-positive versus amyloid-negative patients.^11^ These findings led some clinicians to utilize FTP PET as a diagnostic tool among people with suspected CTE-NC. We therefore conducted this study to critically evaluate the clinical utility of FTP PET among former ASF players and similar populations.

Surprisingly, we were unable to replicate the findings of the prior study because we detected no FTP differences between former football players (even when limited to those with significant neurocognitive complaints) and control participants. An important similarity between these studies is the complete lack of association between FTP uptake and objective neurocognitive functioning, thereby emphasizing the difficulty in explaining different clinical phenotypes using objective diagnostic testing.^42^

It is also noteworthy that race-associated differences were seen in the extent of off-target binding in the hippocampus by the choroid plexus, as reported in other studies.^43-45^ Given that the hippocampus is one of the key memory centers believed to be affected by CTE-NC and therefore worthy of in-depth study in former professional ASF players, data from this study suggest that FTP might be confounded in populations that include Black participants or others with high neuromelanin expression. In aggregate, our data question the clinical utility of FTP PET imaging for the ante-mortem detection of CTE-NC in former ASF players and highlight the need for future work to define clinical evaluation of suspected CTE-NC.

There is considerable interest in whether CTE-NC underlies neurocognitive impairment among people exposed to RHI including former ASF players. The lack of association between FTP uptake and objective neurocognitive testing, now consistent across two independent studies of prior ASF players,^6^ challenges this notion.

There are several possible explanations for this null finding. First, it is possible that FTP is less adequately specific as a tracer for the cerebral p-Tau isoform responsible for CTE-NC^10,46^ than previously proposed and thus failed to identify former ASF players with bona fide CTE-NC. Second, it is possible that acquired cerebral tauopathies with more regional specificity than that typically associated with post-mortem-verified CTE-NC may account for ante-mortem neurocognitive impairment or that FTP may not detect specific trauma-related tau isoforms.^47^ It is also possible that FTP shows only a strong affinity for p-Tau in those with severe disease.^11^

Finally, it is possible that neurocognitive symptoms among former ASF players are driven by alternative forms of pathology and clinical conditions. Disease processes including hypertension, mood disorders, chronic inflammation, and disordered sleep may be underappreciated causes of neurocognitive complaints.^17,48,49^ Future work examining the diagnostic accuracy of newer generation p-Tau PET tracers, the clinical correlates of entorhinal cortex p-Tau uptake, and the role of alternative causes of neurocognitive impairment represent a scientific imperative.^48^

We acknowledge limitations of the current study. First, control participant data were derived from a small sample comprised of white participants. While it is possible that a larger and more racially diverse control sample may have yielded different results, we suspect this is unlikely because an all-white control group, not susceptible to neuromelanin off-target binding, would be more likely to show differences between players and controls.

Second, former ASF players in the current study were slightly younger than those similarly studied,^6^ raising the possibility that FTP-uptake related to ASF participation is age dependent. Sensitivity analyses, however, examining older ASF players in our sample did not reveal differences across age. FTP may only positively identify p-Tau pathology in extreme phenotypes, which likely does not comprise the majority of ASF participants.

Finally, we did not adjust for multiple comparisons because the current study was designed with *a priori* hypotheses regarding FTP and CTE-NC-relevant brain regions of interest. Multiple comparisons adjustment would eliminate the marginally significant relationship seen in the entorhinal cortex in players with football exposure, rendering all results null.

## Conclusions

This study detected little difference in FTP uptake among former professional ASF players, including those with subjective neurocognitive complaints, compared with controls. Among former ASF players, we detected a marginal novel association between professional career duration and FTP uptake in the entorhinal cortex, a brain region associated with age-related p-Tau aggregation and AD neuropathologic change. These findings question the clinical utility of FTP PET for the evaluation of suspected CTE-NC, while highlighting the need for future studies exploring entorhinal cortex p-Tau deposition among persons exposed to RHI.

## Supplementary Material

Supplemental data

Supplemental data
