# Supplementary material for: Tau Positron Emission Tomography and Neurocognitive Function Among Former Professional American-Style Football Players
Source: J Neurotrauma. 2023 Aug 16;40(15-16):1614–24. doi: 10.1089/neu.2022.0454 (PMC10458363; doi:10.1089/neu.2022.0454)
Supplement: Supplemental data [file Supp_TableS1.docx]

**Supplementary Materials**

**eTable 1. Derivation of anatomical regions of interest.**

| ***Anatomical region*** | **Atlas or source** | **Region name in Atlas/source** |
| --- | --- | --- |
| Left Parietal | MNI Structural Atlas left part; threshold 50 | Parietal Lobe |
| Superior Frontal | Harvard Oxford Cortical; threshold 50 | Superior Frontal Gyrus |
| Medial Temporal | Harvard Oxford MTC; threshold 50 | Medial Temporal Cortex |
| Parahippocampus | Subject-specific MRI space | Parahippocampal |
| Hippocampus | Subject-specific MRI space | Hippocampus |
| Amygdala | Subject-specific MRI space | Amygdala |
| Entorhinal cortex | Subject-specific MRI space | Entorhinal |
