# Supplementary material for: Tau Positron Emission Tomography and Neurocognitive Function Among Former Professional American-Style Football Players
Source: J Neurotrauma. 2023 Aug 16;40(15-16):1614–24. doi: 10.1089/neu.2022.0454 (PMC10458363; doi:10.1089/neu.2022.0454)

**eFigure 1. Mean [^18^F]-FTP SUVR maps in former ASF players and controls.** Former ASF players shown in top panel and controls are shown in the lower panel with the cerebellum non vermis serving as reference region. Color gradient indicates [^18^F]-FTP SUVR taken at 80-100 minutes.


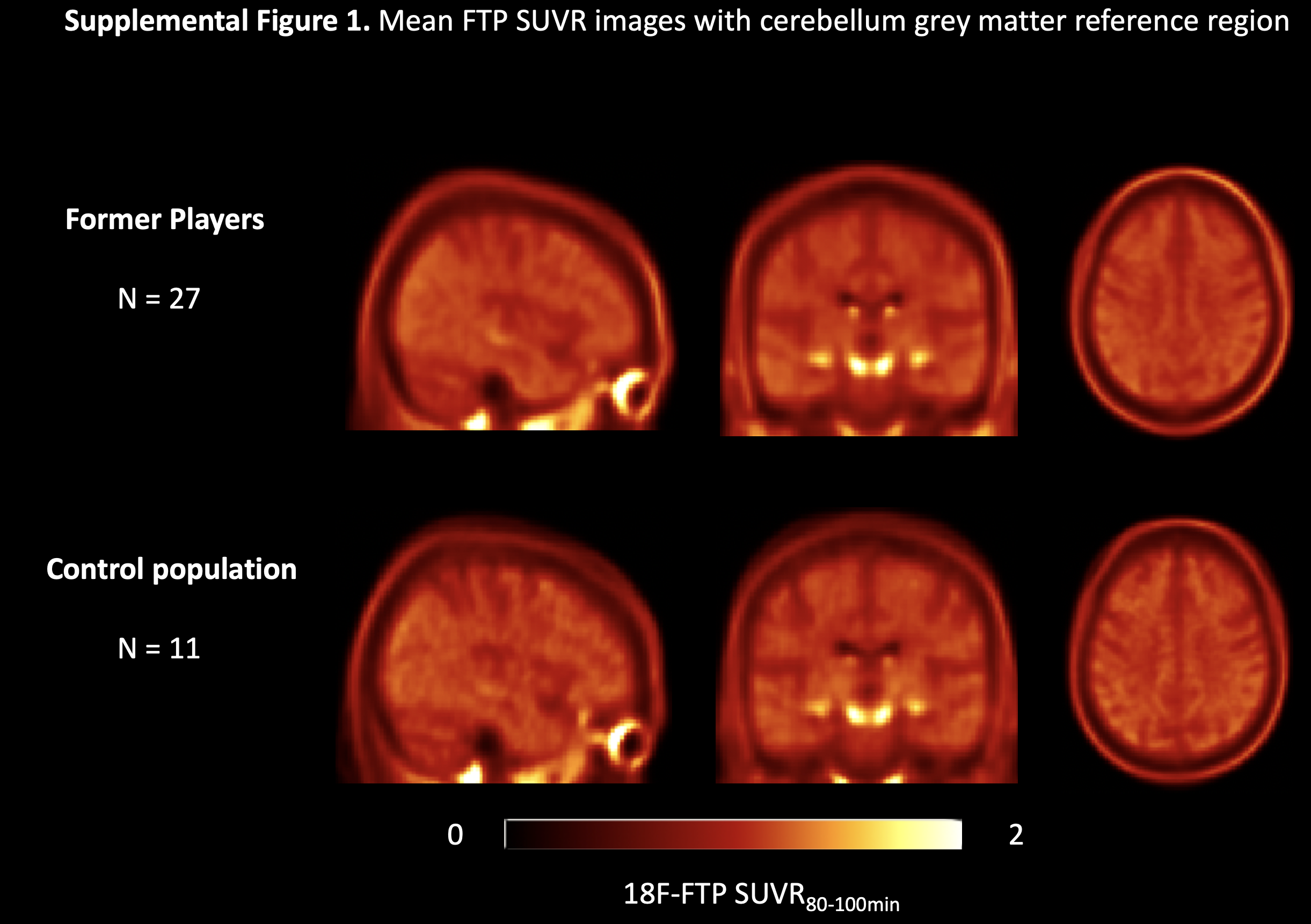

Supplement: Supplemental data [file Supp_FigS1.docx]
